# The Effects and Mechanisms of Continuous 7-Day Hypobaric Hypoxia Exposure on Sleep Architecture in Rats

**DOI:** 10.3390/ijms26114998

**Published:** 2025-05-22

**Authors:** Fang Li, Xianxie Zhang, Anping Ye, Ling Qi, Tianke Huang, Xitai Chen, Maoxing Li, Chengrong Xiao, Yuguang Wang, Yue Gao, Zengchun Ma

**Affiliations:** 1 School of Traditional Chinese Medicine, Guangdong Pharmaceutical University, Guangzhou 510006, China; 15391687885@163.com (F.L.); 18099817982@163.com (L.Q.); huangtianke2022@163.com (T.H.);; 2 Department of Pharmaceutical Sciences, Beijing Institute of Radiation Medicine, Beijing 100850, China; zhangxianxie@163.com (X.Z.); anpingye@163.com (A.Y.);

**Keywords:** plateau insomnia, rat model, wireless EEG telemetry, sleep rhythm and homeostasis

## Abstract

High-altitude environments pose significant risks for insomnia development, which severely compromises both physiological health and occupational performance. To elucidate the mechanisms underlying altitude-induced sleep disruption and establish a validated animal model for therapeutic intervention development, we exposed Sprague-Dawley rats to hypobaric hypoxia (5500 m altitude equivalent: 308 mmHg, 20.37% O_2_, PiO_2_ 8.0 kPa) for 7 days. We employed continuous wireless telemetry to monitor EEG/EMG signals, with concurrent analysis of physiological parameters, blood biochemistry, histopathology, transcriptomics, and protein expression. Quantitative analyses demonstrated decreased caloric intake, transient body mass reduction, and immune-metabolic disturbances. While total sleep duration showed no significant variation, sleep architecture displayed elevated wakefulness periods, reduced active wakefulness, a decreasing trend of slow-wave sleep (SWS), and increased paradoxical sleep (PS) accompanied by attenuated circadian oscillations. The duration of SWS episodes was significantly shortened, indicating a sleep homeostasis imbalance that peaked on day 3. Biochemical profiling revealed reduced levels of antioxidant enzymes, elevated pro-inflammatory cytokines, and hypothalamic–pituitary–adrenal axis activation. Transcriptomic analyses identified the critical involvement of serotonergic/glutamatergic synaptic regulation, lipid metabolism, IL-17 signaling, and cortisol synthesis pathways. Western blot analyses confirmed OX2R upregulation, 5-HT1AR downregulation, and circadian gene dysregulation. Our findings demonstrate that hypobaric hypoxia induces sleep disruption via coordinated mechanisms involving oxidative stress, inflammatory activation, HPA axis hyperactivity, neurotransmitter imbalance, and circadian clock dysfunction, providing a robust preclinical model for mechanistic exploration and therapeutic target identification.

## 1. Introduction

Upon entering high-altitude regions, humans often encounter various sleep disturbances. Millions of individuals annually visit high-altitude regions, and over 80 million people reside permanently above elevations of 2500 m. Acute hypobaric hypoxia exposure induces high-altitude illness (HAI), encompassing clinically significant syndromes such as acute mountain sickness (AMS), high-altitude cerebral edema (HACE), and high-altitude pulmonary edema (HAPE). Studies have shown that the incidence of sleep disorders in individuals rapidly ascending to high altitudes ranges from 71% to 93% [1,2,3]. Typical symptoms include difficulty falling asleep, frequent awakenings, early morning arousal, and persistent fatigue upon waking. These disturbances primarily manifest as a decline in sleep efficiency (SE), while total sleep time (TST) remains unchanged. However, deep non-rapid eye movement (NREM) sleep stages (III and IV) transition into lighter sleep stages (I and II), slow-wave sleep (SWS) decreases, and awakenings become more frequent. Many clinical studies have validated these patterns. For instance, Konrad E. Bloch’s research demonstrated that reductions in slow wave sleep are the most consistent altitude-induced changes in sleep structure identified by visual scoring, yet the underlying mechanisms remain unclear [4,5,6,7]. Therefore, establishing a stable animal model for high-altitude insomnia is crucial for understanding disease characteristics and facilitating drug development.

The development of high-altitude sleep models is constrained by the limitations of simulating hypobaric hypoxia environments and the availability of low-disturbance EEG monitoring equipment. Conventional animal EEG monitoring systems cannot be used within hypobaric hypoxia chambers, and wired monitoring systems often restrict animal movement, potentially interfering with experimental outcomes. With technological advancements, minimally invasive sleep monitoring techniques have emerged, providing new tools for studying high-altitude sleep disturbances [8]. For instance, wireless EEG recording technology [9], non-invasive physiological monitoring devices [10], and miniaturized sensors enable researchers to collect continuous, long-term data under natural sleep conditions, minimizing interference and improving data reliability. These technologies facilitate long-term, continuous EEG monitoring in freely moving rodents, allowing the assessment of sleep structure under special environmental conditions while avoiding stress responses associated with traditional monitoring methods.

In this study, we utilized a DSI wireless physiological telemetry system (HD-S02 Physiological Signal Telemetry Implant, PCR-1 Wireless Signal, Neuroscore Sleep Signal Analysis Software, and Phonema Physiological Signal Acquisition Software, U.S. Data Science Internal Company [DSI]) to systematically examine changes in sleep duration and structure in rats subjected to seven consecutive days of hypobaric hypoxia exposure. We also explored the underlying mechanisms, aiming to establish a stable high-altitude insomnia rat model that provides a foundation for studying disease characteristics and developing therapeutic interventions.

## 2. Results

### 2.1. Changes in Sleep Duration in Rats Following 7 Days of Continuous Hypobaric Hypoxia Exposure

The sleep architecture of rats differs significantly from that of humans in terms of cycle length, REM sleep proportion, NREM sleep stages, sleep–wake rhythm, and sleep requirements [11]. Observations revealed that after entering the hypoxic chamber, rats exhibited dull and dry fur, lethargy, drowsiness, rapid breathing, increased thoracic fluctuations, mental fatigue, curled limbs, reduced activity, and decreased aggression.

**Figure 1:**
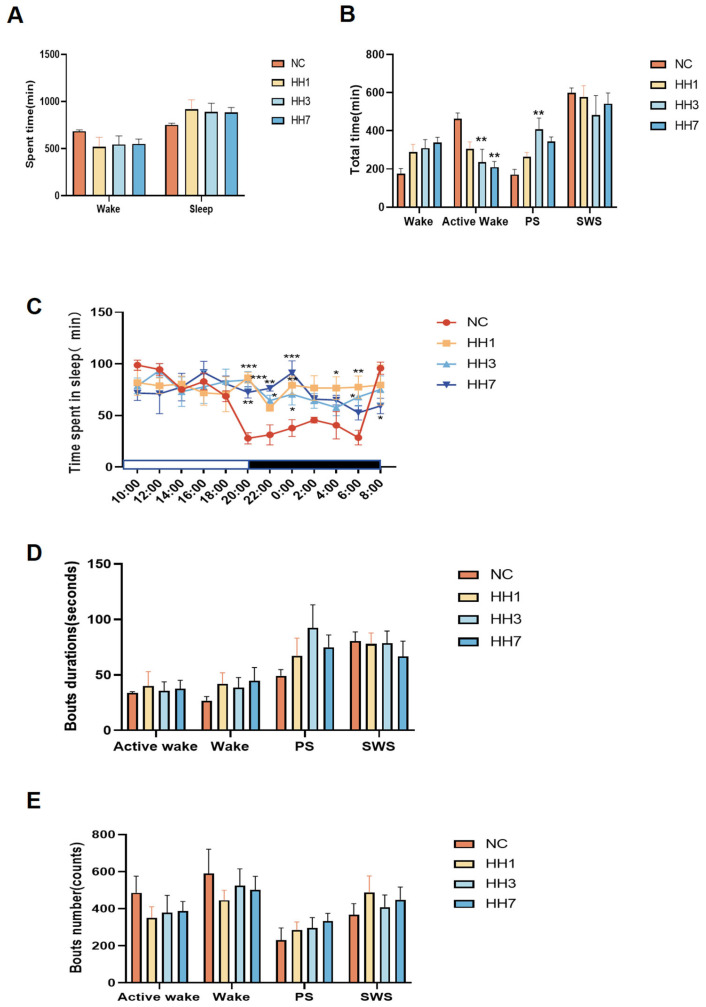
Effect of different hypoxia models on 24 h sleep characteristics in rats. (**A**) Total sleep–wake duration; (**B**) duration of sleep–wake phases (including wakefulness, active wakefulness, heterogeneous sleep, and slow-wave sleep); (**C**) distribution of sleep per 2 h in a 24-h period; (**D**) average duration of each sleep–wake phase segment. (**E**) Number of sleep–wake phase fragments * *p* < 0.05, ** *p* < 0.01, *** *p* < 0.001 vs. control group.

Sleep duration is a fundamental indicator of sleep evaluation, while the distribution of sleep phases (including quiet wake, active wake, paradoxical sleep, and slow-wave sleep) serves as a measure of sleep quality. According to the guidelines set by the American Academy of Sleep Medicine (AASM) [12], DSI telemetry technology was used to analyze specific waveforms (e.g., δ waves and θ waves) to differentiate NREM and REM sleep stages. Analysis of total sleep time, total wake time, and sleep–wake cycle distribution revealed that the total sleep time of rats did not significantly change after entering the hypoxic chamber (Figure 1A). Compared to the normal control group, rats in the hypobaric hypoxia groups exhibited a reduction in active wake time, with significant reductions observed in the 3-day and 7-day hypoxia groups (*p* < 0.01). The duration of PS, indicative of shallow sleep, increased, leading to a decline in sleep quality. In particular, the PS duration in the 3-day hypoxia group was significantly lower than that in the control group (*p* < 0.01) (Figure 1B), suggesting a state of first stress and then adaptation.

Additionally, SWS, indicative of deep sleep, was reduced.

### 2.2. Changes in Sleep Rhythm and Sleep Phases on Days 1, 3, and 7 of Continuous Hypobaric Hypoxia Exposure

Rats exhibit a regular sleep–wake cycle over 24 h, with most total sleep time (SWS + PS) occurring during the dark phase and most total wake (QW + AW) occurring during the light phase. Analysis of the 24-h sleep–wake cycle (12-h dark phase (active period) and 12-h light phase (rest period)) revealed that in the normal control group, the area under the curve (AUC) for sleep duration was larger during the light phase than during the dark phase. However, in the hypoxia model groups, the AUC for sleep duration during the dark phase increased, nearly equaling that of the light phase. Specifically, between 20:00 and 06:00, the AUC for sleep duration was 245.7 in the control group, compared to 450.4, 404.6, and 416.8 in the hypoxia groups on days 1, 3, and 7, respectively, indicating reduced circadian oscillation in sleep rhythms. At specific time points (20:00, 22:00, 00:00, 04:00, and 06:00), sleep duration increased significantly (*p* < 0.05, *p* < 0.01), reflecting abnormal activity and circadian rhythm disruption (Figure 1C).

Analysis of the mean duration of sleep phase segments showed that after hypoxia exposure, the duration of wakefulness and active wakefulness segments increased, while the duration of PS segments also increased, and SWS segments decreased (Figure 1D), suggesting that hypobaric hypoxia reduces sleep continuity. Analysis of the number of sleep phase segments revealed that after entering the hypoxic chamber, the number of wakefulness and wakefulness wake segments decreased, whereas the number of PS and SWS segments increased (Figure 1E), indicating increased sleep fragmentation due to hypobaric hypoxia. Among the different groups, the 3-day hypoxia group exhibited the most pronounced changes in all indicators, showing a state of first stress and then adaptation.

Results from the analysis of sleep duration, rhythm, and phases demonstrated that compared to baseline data, the total sleep–wake duration remained unchanged under hypobaric hypoxia conditions. However, active wakefulness time was significantly reduced, overall sleep–wake quality declined, sleep duration during the active period increased, and sleep during the rest period significantly decreased. Additionally, the overall sleep–wake rhythm showed weakened oscillation.

### 2.3. Sleep Architecture Characteristics on Day 3 of Continuous Hypobaric Hypoxia Exposure

**Figure 2:**
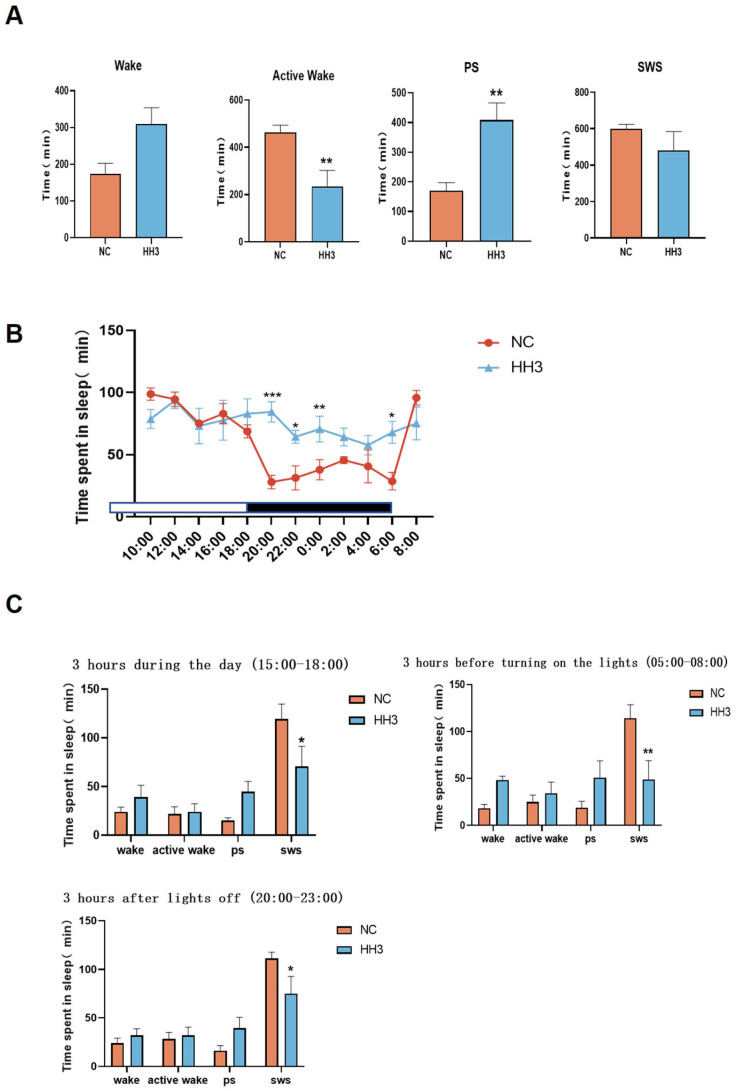
Effects of low-pressure, low-oxygen exposure on 24 h sleep characteristics of rats on day 3. (**A**) Duration of sleep–wake phases (including wakefulness, active wakefulness, heterogeneous sleep, and slow-wave sleep); (**B**) distribution of sleep per 2 h in a 24 h period; (**C**) duration of 3 h of sleep in each stage. *p < 0.05, ** *p* < 0.01, *** *p* < 0.001 vs. control group.

Analysis of the sleep–wake phases (including wakefulness, active wakefulness, paradoxical sleep, and slow-wave sleep) revealed that the 3-day hypobaric hypoxia group exhibited significantly reduced sleep quality. Therefore, we conducted a further comparative analysis between the 3-day hypobaric hypoxia group and the normal control group. The results showed that the active wakefulness duration in the 3-day hypoxia group was significantly reduced (*p* < 0.01), while PS duration was significantly increased (*p* < 0.01), and SWS duration also increased (Figure 2A).

Analysis of the 24 h sleep–wake cycle (12-h dark [active] period and 12-h light [rest] period) showed that, compared to the normal control group, rats in the 3-day hypobaric hypoxia group exhibited abnormal sleep–wake activity (Figure 2B). During the active period, sleep duration significantly increased between 22:00–00:00 and 04:00–06:00 compared to the control group. During the rest period, sleep duration significantly decreased between 18:00 and 20:00 in the 3-day hypoxia group compared to the control group.

To further characterize awakening changes, we analyzed the sleep–wake characteristics of rats during three different 3 h periods: 15:00–18:00 (daytime), 20:00–23:00 (early dark phase), and 05:00–08:00 (early light phase). The results showed that paradoxical sleep duration increased in all three time periods in the 3-day hypoxia group, whereas slow-wave sleep duration significantly decreased (*p* < 0.05) (Figure 2C), with the most pronounced changes observed in the early light phase (*p* < 0.01). These findings suggest that, compared to the NC group, the HH3 group exhibited significantly reduced deep sleep, increased wakefulness, and an overall decline in sleep–wake quality.

### 2.4. Effects of Different Hypobaric Hypoxia Exposure Durations on Physiological and Biochemical Parameters in Rats

No rats died during the experiment. Compared to the control group, rats in the hypoxia model groups showed reduced food and water intake. Their body weight initially decreased on the first day and then gradually increased at a slow rate.

**Figure 3:**
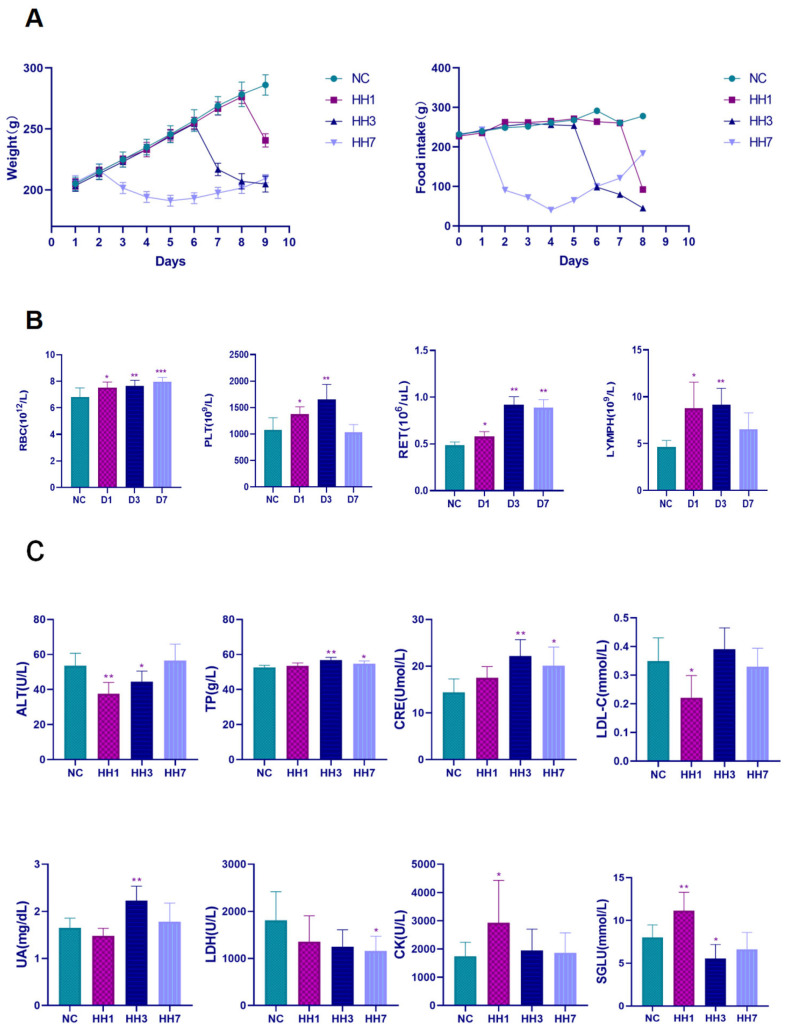
Effects of different hypoxia models on physiological and biochemical indexes. (**A**) Change in percentage of body weight, change in food intake; (**B**) changes in the main indicators of routine blood tests; (**C**) change in serum biochemical markers * *p* < 0.05, ** *p* < 0.01, *** *p* < 0.001 vs. Control group.

Hematological analysis revealed that, compared to the control group, red blood cell (RBC) count, hemoglobin (HGB) concentration, and reticulocyte percentage (RET%) increased with prolonged hypoxia exposure. Platelet count (PLT) and lymphocyte percentage (LYMPH%) initially increased (*p* < 0.01, 3HH vs. control) and then decreased over time, showing no significant difference between the 7-day hypoxia group and the normal group. White blood cell (WBC) count and monocyte percentage (MONO%) fluctuated with immune responses. In the 1-day hypoxia group, WBC and MONO% levels initially increased significantly (*p* < 0.05) but subsequently decreased with prolonged hypoxia, showing no significant difference in the 7-day hypoxia group. However, the neutrophil percentage (NEUT%) did not show significant changes across groups (Figure 3B). These findings indicate that hypobaric hypoxia significantly affects white blood cell, red blood cell, and platelet indices in rats.

Biochemical marker analysis showed that CK and LDH are key indicators of myocardial injury, CREA and UA reflect renal function, ALT and TP indicate liver damage, while LDL-C and SGLU are markers of lipid metabolism. We measured serum levels of CK, LDH, CREA, UA, ALT, TP, LDL-C, and SGLU (Figure 3C). Compared to the control group, serum CK and SGLU levels significantly increased after 1 day of hypoxia (*p* < 0.05), while ALT and LDL-C levels significantly decreased (*p* < 0.05), with no significant changes in other parameters. In the 3-day hypoxia group, serum CREA, UA, and TP levels significantly increased (*p* < 0.05), while ALT and SGLU levels significantly decreased (*p* < 0.05). No other significant differences were observed. In the 7-day hypoxia group, TP and CREA levels showed a significant increase (*p* < 0.05), while LDH levels significantly decreased (*p* < 0.01). These findings suggest that hypobaric hypoxia causes fluctuations in markers of cardiac, hepatic, and renal function, as well as metabolic parameters, leading to significant physiological disturbances that vary over time.

### 2.5. Effects of Different Hypobaric Hypoxia Exposure Durations on Brain Tissue Pathology

**Figure 4:**
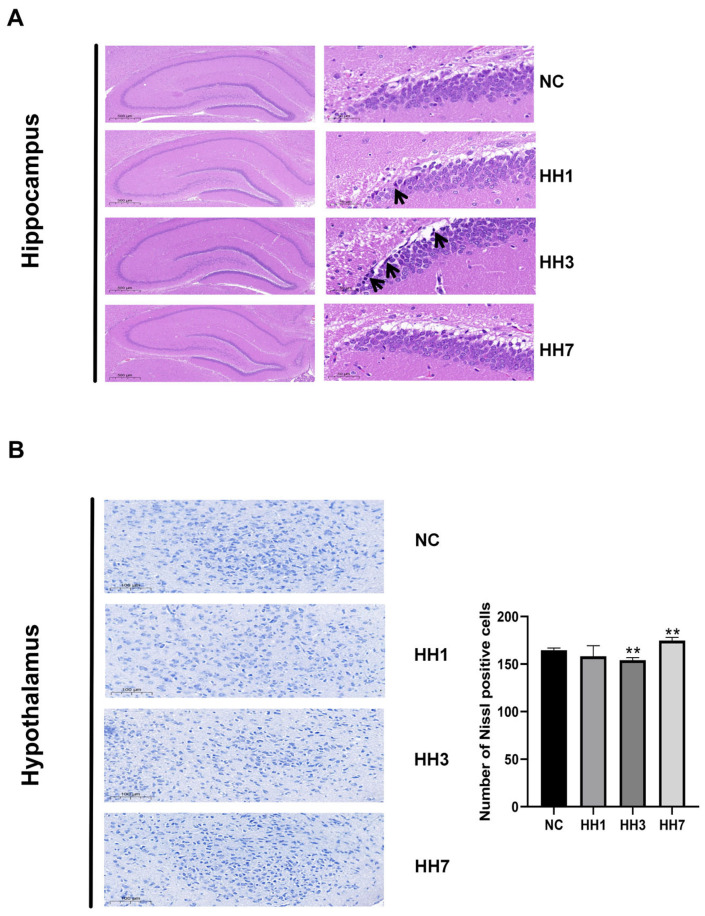
Effects of different hypoxic models on hippocampal and hypothalamic pathology in rats. (**A**) Hippocampal dentate gyrus (DG) region (2×, 20× magnification). Arrows indicate neuronal collapse. (**B**) Nissl staining of hypothalamic tissue (10× magnification). ** *p* < 0.01 vs. control group.

To assess the effects of different hypoxia exposure durations on neuronal damage, we performed H&E and Nissl staining on hippocampal and hypothalamic tissues. In the NC group, pyramidal cells in the hippocampal pyramidal layer were tightly arranged, morphologically intact, and exhibited clear structures, abundant cytoplasm, and distinct nuclei. However, in the hypoxia groups, varying degrees of abnormalities were observed, including loose pyramidal cell arrangement, neuronal shrinkage, deepened staining, and unclear nuclear and cytoplasmic boundaries, though no inflammatory cell infiltration was detected. The most severe damage was observed in the 3-day hypoxia group (Figure 4A).

Nissl staining results revealed neuronal damage and degeneration in the hypothalamic DG region across all hypoxia groups compared to the control group. Nissl bodies were sparsely arranged, with significantly reduced volume and numbers (*p* < 0.01) (Figure 4B). The most severe damage was also observed in the 3-day hypoxia group, where neurons exhibited fragmentation and dissolution.

### 2.6. Effects of Different Hypobaric Hypoxia Exposure Durations on Hypothalamic Transcriptomics and Bioinformatics Analysis

**Figure 5:**
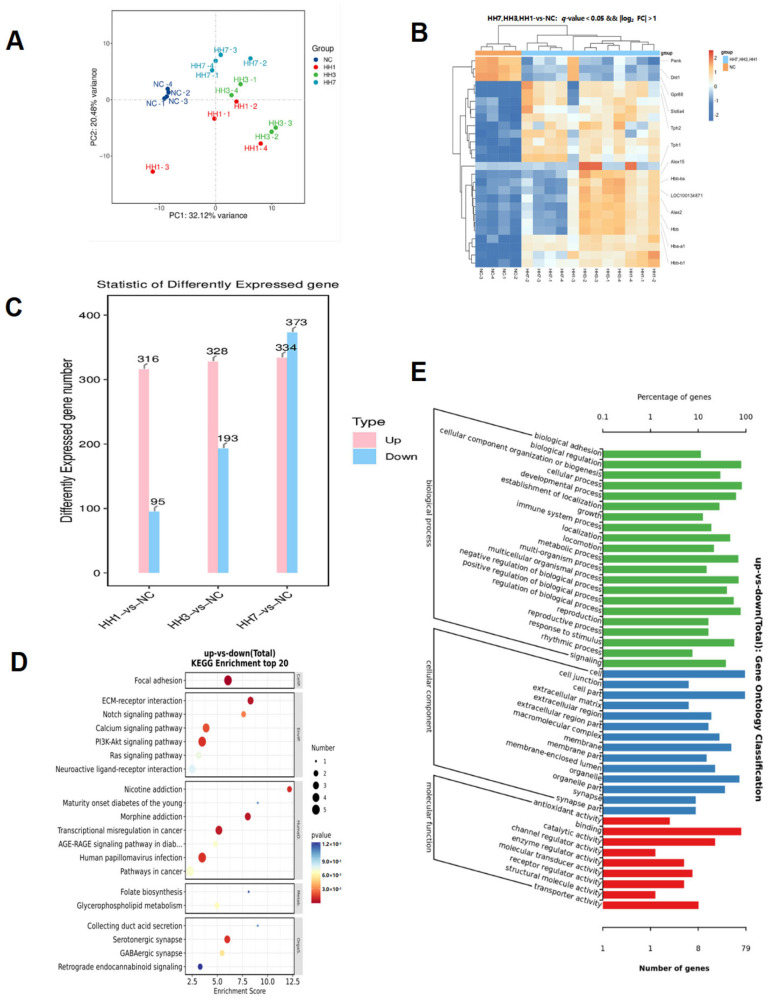
(**A**). Clustering of transcriptomes using Uniform Manifold Approximation and Projection (UMAP). (**B**). Clustering analysis of differentially expressed genes (DEGs). (**C**) Quantification of significantly up- and downregulated genes between the normal control group (NC) and the hypoxia and low-pressure groups of different durations (SD). (**D**) Histogram of KEGG enrichment analysis of common target (Cellular processes, Environmental processing, Human diseases, Metabolism, Organismal System). (**E**). Histogram of GO enrichment analysis.

Principal component analysis (PCA) was used to explore transcriptomic differences in the hypothalamus of rats exposed to different durations of hypoxia. The results showed clear separation between groups, with strong intra-group clustering, indicating consistent gene expression changes within groups and reliable inter-group differences (Figure 5A).

A heatmap of differentially expressed genes (DEGs) showed significant differences between the NC and hypoxia groups (Figure 5B). The number of DEGs increased with prolonged hypoxia exposure. Compared to the control group, 511 DEGs were identified in the 1-day hypoxia group, 521 in the 3-day hypoxia group, and 707 in the 7-day hypoxia group. Among these differentially expressed genes, PRPH2 and S100A11 exhibited significant upregulation, with their retinal functional alterations potentially disrupting photic signal transduction, thereby inducing melatonin secretion abnormalities that may compromise sleep regulation. Notably downregulated genes, including MLIP and TGIF2, demonstrated functional interactions with the PI3K/AKT signaling pathway, suggesting their potential regulatory roles in sleep modulation and that these genes may be key targets of hypoxia-induced damage (Figure 5C).

KEGG enrichment analysis was performed to identify biological functions and pathways associated with these DEGs. The top 20 enriched pathways (based on *p*-values) were visualized, revealing that the DEGs were primarily involved in serotonergic synapses, glutamatergic synapses, lipid metabolism, IL-17 signaling, cortisol synthesis and secretion, and other related signaling pathways (Figure 5D).

GO analysis indicated that cellular components were mainly associated with cell membranes, synapses, and adhesion. Molecular functions were related to enzymatic activity, molecular transport, and receptor binding, while biological processes were enriched in circadian rhythm regulation and immune system processes (Figure 5E). These findings suggest that key research areas for hypoxia-induced insomnia in high-altitude environments should focus on oxidative stress and inflammation, neurotransmitter synthesis, cortisol expression changes, and circadian rhythm regulation.

### 2.7. Effects of Different Durations of Hypobaric Hypoxia Exposure on Oxidative Stress and Inflammatory Factors

High altitude compensates for the decrease in oxygen bioavailability by altering the release of inflammatory factors and enzyme activity, among other things. Oxidative stress damages brain regions involved in sleep regulation, promotes inflammation, disrupts circadian rhythms, and interferes with the sleep–wake cycle. To evaluate oxidative stress and inflammation levels, we measured serum levels of relevant enzymes and cytokines using ELISA.

**Figure 6:**
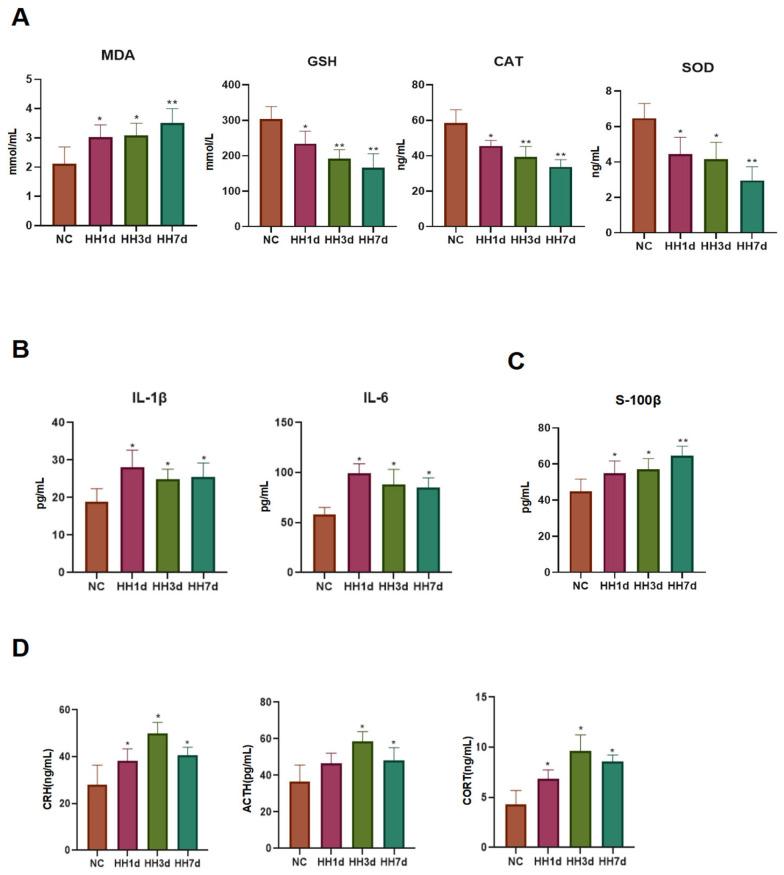
Hypobaric hypoxia induces oxidative stress and inflammatory responses, reduces blood–brain barrier (BBB) permeability, and activates the hypothalamic–pituitary–adrenal (HPA) axis in rats. (**A**) Effects of different durations of hypobaric hypoxia exposure on serum oxidative stress markers: MDA, GSH, CAT, and SOD activity. (**B**) Impact of varying hypobaric hypoxia durations on pro-inflammatory cytokine levels: IL-6 and IL-1β. (**C**) Serum levels of S-100β, a biomarker of BBB permeability. (**D**) Alterations in HPA axis-related hormone levels (CORT and ACTH) under different hypobaric hypoxia durations. * *p* < 0.05, ** *p* < 0.01 vs. control group.

As shown in Figure, compared to the control group, rats exposed to prolonged hypobaric hypoxia exhibited significantly lower levels of SOD, CAT, and GSH (*p* < 0.05). Meanwhile, MDA levels were significantly elevated (*p* < 0.05, Figure 6A), indicating the occurrence of oxidative stress under hypoxic conditions. In addition, IL-1β and IL-6 levels were significantly increased (*p* < 0.05, Figure 6B), suggesting that hypoxia induced inflammatory responses. These findings indicate that hypobaric hypoxia alters the release of inflammatory factors and enzymatic activity, which may contribute to changes in sleep patterns.

### 2.8. Effects of Different Durations of Hypobaric Hypoxia Exposure on Blood–Brain Barrier Permeability and the HPA Axis

Changes in blood–brain barrier (BBB) permeability can alter the brain’s microenvironment, thereby affecting neural activity related to sleep–wake regulation. The HPA axis plays a critical role in stress responses. Activation of the HPA axis leads to cortisol release, which affects circadian rhythms and increases BBB permeability, allowing inflammatory factors to enter the brain, exacerbating neuroinflammation, and disrupting sleep regulation.

To assess BBB permeability and HPA axis activity, we measured serum levels of BBB marker S-100B and HPA-related hormones, including the corticotropin-releasing hormone (CRH), the adrenocorticotropic hormone (ACTH), and corticosterone (CORT), using ELISA.

As shown in Figure 6, compared to the control group, serum S-100B levels were significantly elevated in the hypoxia model groups (*p* < 0.05, Figure 6C), indicating increased BBB permeability. Additionally, CRH, ACTH, and CORT levels were significantly elevated (*p* < 0.05, Figure 6D) in the hypoxia-exposed rats, confirming HPA axis activation. These findings suggest that hypobaric hypoxia affects BBB integrity and HPA axis function, potentially contributing to sleep disturbances.

### 2.9. Effects of Different Durations of Hypobaric Hypoxia Exposure on the Orexin System

The orexin system plays a key role in sleep–wake regulation, where increased orexin-A levels promote wakefulness. By activating two G protein-coupled receptors (OXR1 and OXR2), OXR2-mediated signaling demonstrates greater functional specificity in sleep homeostasis compared to OXR1. To assess the impact of hypobaric hypoxia on orexin receptor 2 (OX2R), we examined OX2R protein expression in the hypothalamus.

**Figure 7:**
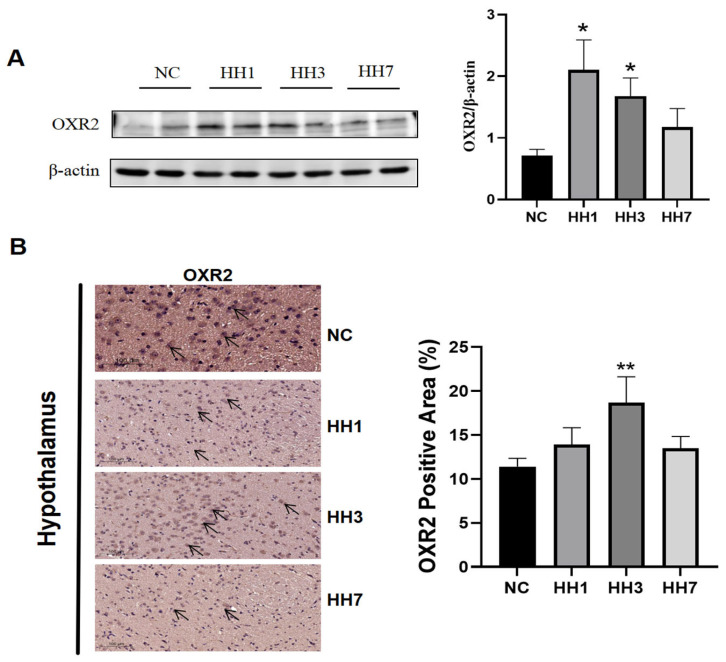
Effect of different hypobaric hypoxia durations of exposure on the orexin system. (**A**) Relative expression levels of OXR2 protein in hypothalamic tissues by Western blotting; (**B**) Immunohistochemical staining results for OXR2 in the hypothalamus. * *p* < 0.05, ** *p* < 0.01 vs. NC group.

As shown in Figure 7, Western blot (WB) and immunohistochemical staining were used to detect OX2R levels. The WB results showed that, compared to the control group, OX2R protein expression was significantly upregulated in the hypoxia model groups (*p* < 0.05, Figure 7A). Immunohistochemical staining further revealed a significant reduction in the positive staining area in the hypoxia groups (*p* < 0.05, Figure 7B). These results suggest that hypobaric hypoxia may regulate sleep by modulating OX2R expression.

### 2.10. Effects of Different Durations of Hypobaric Hypoxia Exposure on Sleep–Wake-Related Neurotransmitters and Hypothalamic 5-HTR1A Expression

**Figure 8:**
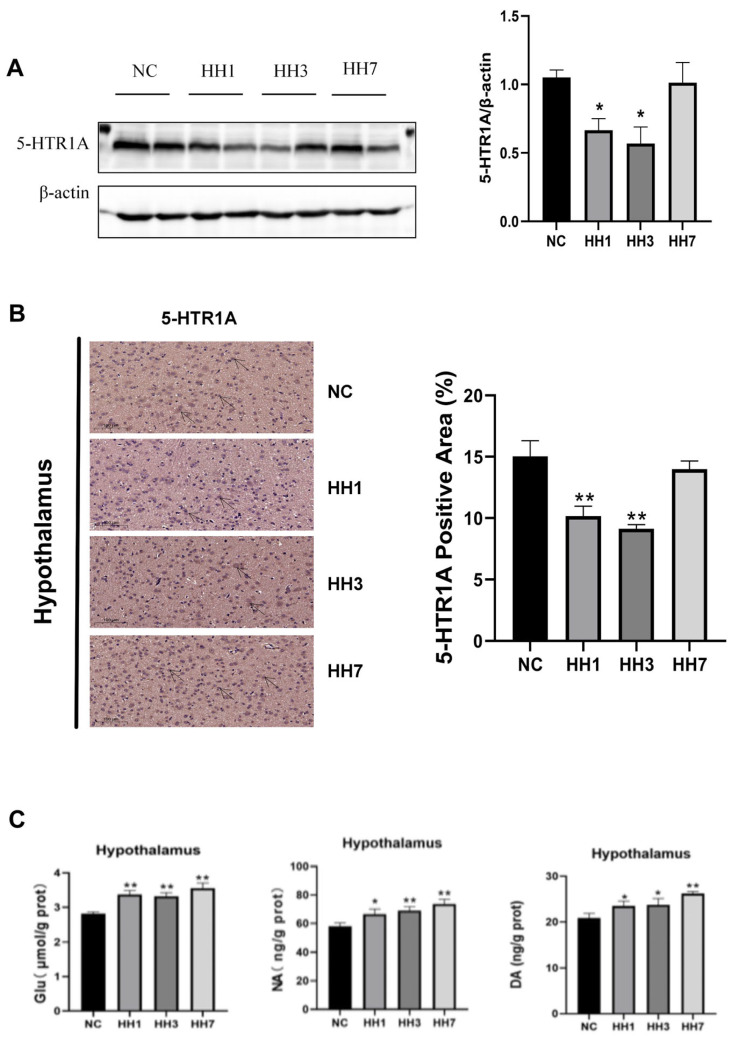
Effects of different hypobaric and hypoxic durations of exposure on hypothalamic 5-HTR1A protein expression and changes of sleep–wake related neurotransmitters. (**A**) Relative expression levels of 5-HTR1A protein in hypothalamic tissues by Western blotting; (**B**) immunohistochemical staining results for 5-HTR1A in the hypothalamus; (**C**) changes in sleep–wake-related neurotransmitters of hypothalamus. * *p* < 0.05, ** *p* < 0.01 vs. NC group.

5-hydroxytryptamine receptor 1A (5-HTR1A) modulates serotonin (5-HT) release and signal transduction, which affects the sleep–wake cycle. Disruptions in serotonin signaling can lead to heightened wakefulness and impaired sleep. To investigate this, we assessed hypothalamic 5-HTR1A protein expression using WB and immunohistochemical staining. Alterations in the concentrations of other neurotransmitters were quantitatively analyzed using enzyme-linked ELISA. As shown in Figure 8, the WB results indicated that 5-HTR1A protein levels were significantly decreased in the hypoxia model groups compared to the control group (*p* < 0.05, Figure 8A). Immunohistochemical staining further showed that the area of 5-HTR1A positive staining in the hypoxia group was significantly reduced (*p* < 0.05, Figure 8B). The expression levels of Glu, NA, DA, and other related arousal neurotransmitters were significantly increased (*p* < 0.01, *p* < 0.05, Figure 8C). These findings suggest that hypobaric hypoxia may impair sleep by downregulating 5-HTR1A in the hypothalamus and increasing the expression of pro-wakefulness-related neurotransmitters.

### 2.11. Effects of Different Durations of Hypobaric Hypoxia Exposure on Transcription-Translation Feedback Loop-Related Proteins

The mammalian circadian rhythm is regulated by a highly conserved transcription-translation feedback loop (TTFL), composed of core clock genes. These include positive regulators (CLOCK, BMAL1) and negative regulators (PER, CRY), which drive daily molecular oscillations, maintaining sleep homeostasis and circadian rhythms.

To assess changes in circadian regulatory proteins, we performed immunohistochemical staining to detect CLOCK protein levels and WB to measure hypothalamic levels of CLOCK, BMAL1, PER2, and CRY2.

**Figure 9:**
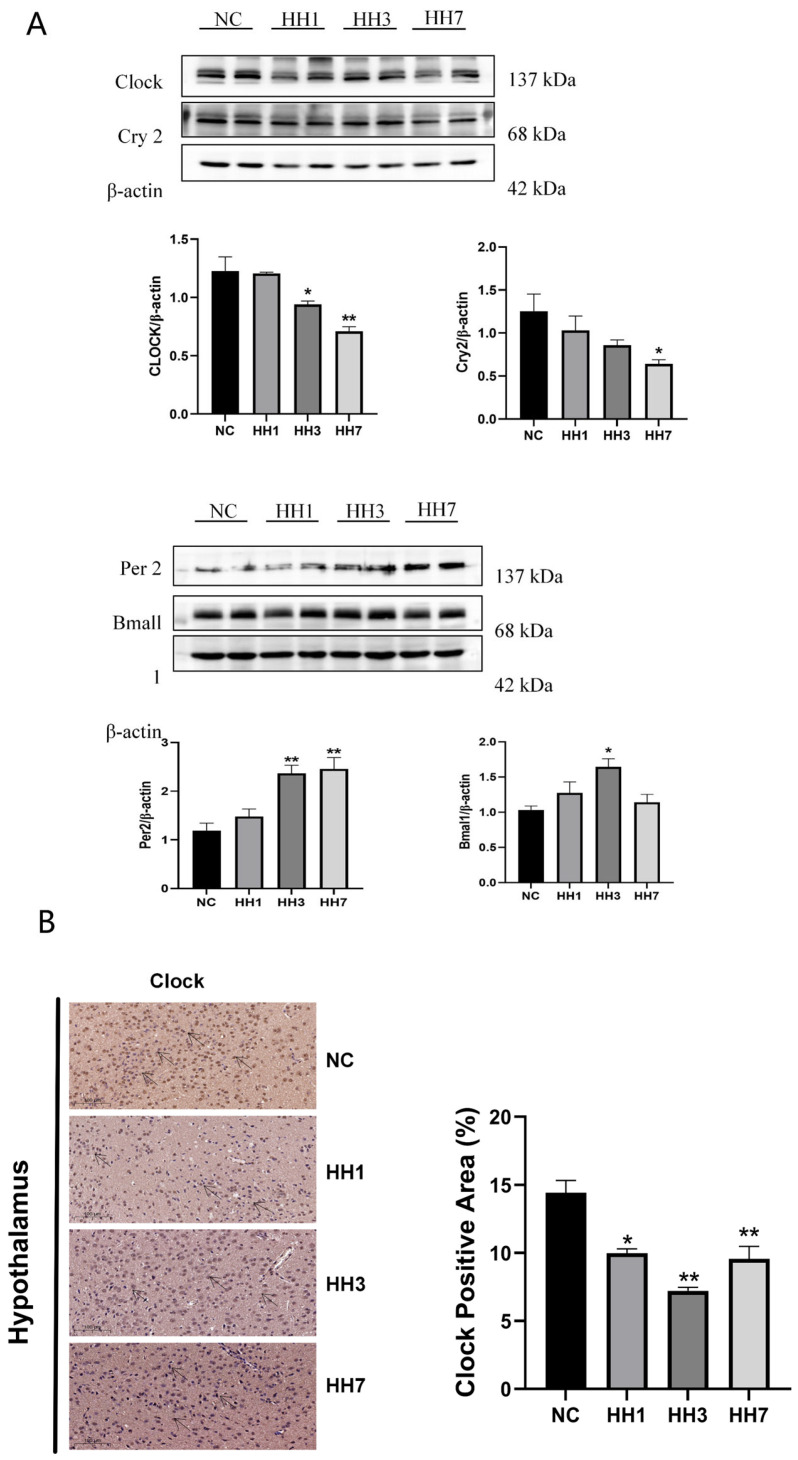
Effects of different durations of hypobaric and hypoxic exposure on the expression of hypothalamic circadian rhythm-related proteins. (**A**) Expression of circadian rhythm-related proteins and hypothalamic clock proteins. (**B**) Immunohistochemical staining results for CLOCK in the hypothalamus. * *p* < 0.05, ** *p* < 0.01 vs. NC group.

As shown in Figure 9, the WB results indicated that, compared to the control group, CLOCK and CRY2 protein levels were significantly decreased in the hypoxia model groups (*p* < 0.05), whereas BMAL1 and PER2 protein levels were significantly increased (*p* < 0.05, Figure 9). Immunohistochemical staining further showed a significant reduction in the hypoxic group’s clock-positive staining area (*p* < 0.05, Figure 9A). These results suggest that hypobaric hypoxia may regulate sleep by reducing CLOCK and CRY2 expression while increasing BMAL1 and PER2 levels.

## 3. Discussion

The impact of high-altitude hypobaric hypoxia on sleep is multifaceted, affecting bioelectrical activity, circadian rhythms, and sleep-related cognitive functions. Establishing a reliable high-altitude insomnia model is essential for advancing disease research and therapeutic development [13,14,15]. This study focused on modeling conditions mimicking 5500 m altitude exposure for varying durations and employed a low-disturbance wireless physiological telemetry system, which allowed for the natural observation of sleep disturbances under hypoxic conditions [16]. Our findings confirmed that hypobaric hypoxia exposure in rats follows an initial stress response leading to adaptation, with the most severe sleep disruptions occurring on the third day. The successful establishment of a high-altitude insomnia rat model provides a foundation for further research on altitude-related sleep disorders and drug development.

There are significant differences between rat and human sleep patterns. Rats are nocturnal, with more fragmented sleep cycles and shorter sleep episodes compared to humans [17]. However, despite differences in sleep structure and behavioral manifestations, both species share clearly defined wakefulness (WAKE), non-rapid eye movement (NREM), and rapid eye movement (REM) sleep stages, with highly similar electroencephalographic (EEG) characteristics [18]. Zhang Wenhui [19] established a stress-induced insomnia model using rats; secondly, the sleep regulation mechanisms of the two are also very similar. S Majumdar [20] studied the sleep mechanism by observing the morphological changes in rat brain neurons after rapid-eye-movement sleep deprivation. These findings suggest that rat models can effectively reflect human sleep structures and quality, making them valuable tools for investigating high-altitude insomnia mechanisms and facilitating drug discovery.

High-altitude insomnia differs from plain-level insomnia in its etiology and clinical manifestations. While the latter results from social, behavioral, and physiological factors, high-altitude insomnia is primarily induced by hypobaric hypoxia [6,21]. Clinically, Panjwani [22] found that individuals acutely exposed to high-altitude environments exhibited increased light NREM sleep (stages 1 and 2) and decreased deep NREM sleep (stages 3 and 4), consistent with our animal study findings. Our study demonstrated that hypoxia-induced insomnia in rats is characterized by unchanged total sleep duration, reduced deep sleep, increased light sleep, disrupted sleep rhythms, and sleep fragmentation. This suggests that our model effectively simulates human sleep patterns under hypobaric hypoxia conditions.

The mechanisms underlying high-altitude insomnia appear complex. Ray et al. [23] proposed that hypoxia disrupts neurotransmitter synthesis in specific brain regions (such as the prefrontal cortex and brainstem), leading to imbalances that ultimately disturb sleep rhythms. Calderon-Jofre [24] reported that melatonin levels increased in individuals exposed to high-altitude hypoxia. In this study, we also investigated neurotransmitter alterations. For example, the orexin system, a key neurotransmitter system involved in regulating the sleep–wake cycle, originates from orexin (ORX)-producing neurons on the lateral side of the hypothalamus. These neurons mediate different physiological effects by activating OX2R. OX2R activation promotes wakefulness, and its selective inhibition leads to increased NREM sleep and decreased REM sleep [25,26]. Our results showed significantly increased OX2R expression in hypoxia-exposed rats, along with an increase in REM sleep. Additionally, wakefulness-promoting neurotransmitters, including noradrenaline (NA) from the locus coeruleus, dopamine (DA) from the ventral tegmental area and substantia nigra, and glutamate (Glu), were all upregulated under hypoxia [27,28,29,30]. Moreover, 5-HTR1A activation in the raphe nucleus suppresses serotonergic neuron activity, indirectly promoting sleep initiation. Hypoxia significantly reduced 5-HTR1A expression, thereby disrupting the hypothalamic rhythm center and affecting the sleep–wake cycle [31]. These findings highlight a complex network involving synaptic modulation, signal transduction, and neurotransmitter interactions.

Our study also found that prolonged hypobaric hypoxia exposure resulted in changes in various physiological functions of the body, including myocardial damage, kidney function and metabolic abnormalities, decreased SOD and antioxidant enzyme levels, increased MDA levels, and oxidative stress induction. IL-1β and IL-6, common pro-inflammatory cytokines, were elevated under hypoxia and oxidative stress conditions, leading to insomnia and circadian rhythm disturbances [32]. Disruptions in circadian rhythms can further alter redox homeostasis, increasing reactive oxygen and nitrogen species, which may in turn influence circadian oscillations. Oxidative stress markers such as MDA and antioxidant enzymes exhibit circadian rhythmicity controlled by clock genes [33,34]. Srinivasan Periasamy [35] demonstrated that oxidative stress and inflammation levels increase in both animals and humans following sleep deprivation.

Additionally, hypoxia-induced hyperactivation of the HPA axis may contribute to sleep disturbances. HPA overactivation is known to increase wakefulness and reduce sleep quality [36,37]. The HPA axis is activated via CRH release from the hypothalamus, followed by ACTH secretion from the pituitary and cortisol release from the adrenal cortex [38]. Cortisol secretion follows a distinct circadian pattern, peaking in the morning to facilitate wakefulness and reaching its nadir at night to promote sleep. The dysregulation of this system reduces SWS and increases wakefulness. Prolonged CRH elevation and hypoxia-induced BBB permeability changes allow peripheral inflammatory cytokines such as IL-6 and TNF-α to enter the brain, further activating the HPA axis and creating a positive feedback loop that disrupts sleep architecture [39,40,41]. These findings suggest that oxidative stress and inflammation directly contribute to weakened circadian rhythms, while excessive HPA axis activation mediates sleep disturbances under hypoxia.

Moreover, our study found that hypoxia-exposed rats exhibited nearly equal total sleep durations during light and dark phases, indicating a disruption of circadian rhythms. Acute hypoxia exposure dampened body temperature and activity rhythms, suggesting that circadian rhythms allow organisms to predict and adapt to environmental changes over approximately 24 h cycles [42,43]. In mammals, the master circadian pacemaker resides in the suprachiasmatic nucleus (SCN) of the hypothalamus, coordinating physiological processes accordingly [44,45]. At the molecular level, core clock genes consist of BMAL1, CLOCK, PER, and CRY, forming a self-sustaining transcription-translation feedback loop (TTFL) that regulates daily molecular oscillations [46]. During the day, CLOCK-BMAL1 activates Per and Cry transcription, while at night, the PER-CRY complex enters the nucleus to inhibit CLOCK-BMAL1, completing the feedback loop [47,48]. Thus, hypoxia may modulate sleep patterns through CLOCK and other core circadian genes, weakening negative feedback regulation and perpetuating circadian disruptions.

In this study, we systematically investigated the sleep patterns and mechanisms underlying hypoxia-induced insomnia in rats. Our findings suggest that hypoxia affects multiple tissues and organs, offering insights into potential therapeutic targets for altitude-induced insomnia. However, certain limitations remain. Future research should further explore neuronal electrophysiological abnormalities (e.g., ion channel dysregulation and synaptic plasticity alterations) and neuroimmune disturbances (e.g., microglia-mediated neuroimmune dysfunction) in the context of altitude-related sleep disorders.

## 4. Materials and Methods

### 4.1. Chemicals and Reagents

Creatine kinase (CK), creatine kinase-MB (CK-MB), urea (UREA), total protein (TP), aspartate aminotransferase (AST), triglycerides (TRIGL), low-density lipoprotein cholesterol (LDLC-3), lactate (LACT2), lactate dehydrogenase (LDHI2), creatinine (CREJ2), glucose (SGLU3), cholesterol (CHO2I), and alanine aminotransferase (ALT) were purchased from Roche Diagnostics (Mannheim, Germany). Radioimmunoprecipitation assay (RIPA) lysis buffer and BCA protein assay kits were obtained from Beyotime Biotechnology (Shanghai, China). Primary antibodies against β-actin, BMAL1, Clock, PER2, and CRY2, as well as horseradish peroxidase (HRP)-conjugated goat anti-rabbit IgG, were purchased from Abcam (Cambridge, UK). Isoflurane was acquired from Shenzhen Ruiwo De Life Technology Co., Ltd. (Shenzhen, China) Malondialdehyde (MDA), glutathione (GSH), superoxide dismutase (SOD), tumor necrosis factor-α (TNF-α), interleukin-6 (IL-6), and rat interleukin-1β (IL-1β) enzyme-linked immunosorbent assay (ELISA) kits were obtained from Jiangsu Meimian Biotechnology Co., Ltd. (Nantong, China).

### 4.2. Wireless Telemetry Sleep Monitoring System

The DSI wireless EEG physiological monitoring system (HD-S02 physiological telemetry implant, PCR-1 wireless signal receiver, Neuroscore sleep analysis software (NeuroScore3.0), and Phonema physiological signal acquisition software (Ponemah6.5)) was purchased from Data Sciences International (DSI, 119 14th St NW Suite 100 St. Paul, MN 55112 USA). A Model 68003 stereotaxic instrument and R550 multi-channel small animal anesthesia machine were obtained from Shenzhen Ruiwo De Life Technology Co., Ltd., and a Model 5424R refrigerated high-speed centrifuge was acquired from Eppendorf (Wesseling-Berzdorf, Germany).

### 4.3. Animal Model

Male Sprague–Dawley (SD) rats (180–200 g, SPF grade) were obtained from Beijing Vital River Laboratory Animal Technology Co., Ltd. (SCXK [Beijing, China] 2021-0011). The animals were housed in the animal facility of the Academy of Military Medical Sciences, with controlled temperature (22 ± 2 °C), humidity (50 ± 10%), and a 12 h/12 h light-dark cycle (lights on at 08:00, lights off at 20:00), with free access to food and water. The animal experiments were approved by the Institutional Animal Care and Use Committee (IACUC) of the Academy of Military Medical Sciences (Approval No. IACUC-DWZX-2023-541).

1. Experiment 1: Sleep Duration and Structure in Rats

Six SD rats were used for wireless EEG detection. Baseline sleep data were collected under normoxic conditions before exposure to a simulated 5500 m hypobaric hypoxia environment for seven days, during which sleep data were continuously recorded.

2. Experiment 2: Mechanisms Underlying Hypobaric Hypoxia-Induced Insomnia

Forty SD rats were randomly divided into four groups: control (NC), 1-day hypoxia (HH1), 3-day hypoxia (HH3), and 7-day hypoxia (HH7). All rats were acclimated for three days prior to the experiment. Subsequently, the 7-day hypoxia group was housed in a hypoxic chamber for continuous hypoxia exposure throughout the experimental period. The 3-day hypoxia group was exposed to hypoxic conditions starting on day 7 of the experiment for a duration of three days, while the 1-day hypoxia group underwent hypoxia exposure commencing on day 9 for 24 h. Except for the control group, all rats were placed in a simulated hypobaric hypoxia chamber (5500 m altitude, DWC5-III C model, Fenglei, Shanghai, China). Control animals were housed under normoxic conditions in a separate room with identical temperature, humidity, and lighting conditions.

### 4.4. Establishment of the Wireless Sleep Monitoring System in Rats

After one week of acclimatization, SD rats underwent implantation surgery for wireless telemetry devices. Rats were anesthetized with isoflurane (1.5% maintenance in 2 L/min oxygen). The surgical area was shaved, disinfected with iodophor and 75% ethanol, and the animal was secured in a stereotaxic frame. A 2 cm longitudinal incision was made on the scalp, exposing the skull. Two burr holes were drilled at coordinates 3 mm anterior to bregma and 2.8 mm posterior to lambda, with electrodes implanted under the dura mater. Electrodes were secured with dental cement, and the transmitter was implanted subcutaneously in the back. The incision was sutured, and rats were placed in a warm environment until recovery. Postoperative care included intraperitoneal administration of penicillin for three days to prevent infection.

After a two-week recovery period, rats were divided into normoxic and hypobaric hypoxic groups. Each rat first underwent a 24 h baseline EEG recording under normoxic conditions, followed by continuous seven-day recordings in the hypobaric hypoxia chamber. EEG recordings were collected at 08:00 daily, and data were analyzed using NeuroScore 3.1.1 software. Sleep–wake states were categorized into wakefulness (wake), active wakefulness (active wake), paradoxical sleep (PS), and slow-wave sleep (SWS), with measurements including bout numbers, bout mean duration, and total time.

### 4.5. Physiological Assessments

During the experiment, the body weight and food intake of the rats were recorded daily. After successful model establishment, the rats were anesthetized via intraperitoneal injection of 3% sodium pentobarbital (45 mg/kg) at 8 p.m., and whole blood samples were collected from the abdominal aorta. A portion of the blood was stored in anticoagulant tubes for hematological analysis, while the remainder was placed in non-anticoagulant tubes for biochemical assays. The non-anticoagulated blood samples were stored at 4 °C for 30 min and centrifuged at 1500 rpm for 15 min at 4 °C, and the resulting serum was collected and stored at −80 °C. Hematological parameters were measured using a hematology analyzer (XN-1000V, Sysmex, Kobe, Japan), while serum biochemical indices were analyzed using an automated biochemical analyzer (Cobas C 311, Roche, Mannheim, Germany).

### 4.6. Histological Analysis

Following blood collection from the abdominal aorta, the brain was carefully excised, dissected, and fixed in 4% paraformaldehyde. The fixed tissues were subsequently dehydrated and embedded in paraffin, and 5 µm-thick paraffin sections were prepared before deparaffinization and rehydration.

#### Hematoxylin-Eosin (H&E) and Nissl Staining

The prepared paraffin sections were immersed in hematoxylin solution for 10–15 min, rinsed with distilled water, and then stained with eosin solution for 15 s, followed by another rinse. The sections were sequentially dehydrated using 75%, 85%, 95%, and 100% ethanol for 2 min at each step, cleared with xylene, and mounted with neutral resin. After air drying at room temperature, the slides were observed under a microscope. For Nissl staining, paraffin sections were incubated in 1% cresyl violet solution at room temperature for 30 min, washed with distilled water, and subsequently dehydrated and cleared as described above.

### 4.7. Immunohistochemical Staining

Fixed hippocampal and hypothalamic paraffin-embedded tissue sections were subjected to antigen retrieval by heating in citrate buffer (pH 6.0) using a microwave oven. After boiling, the sections were incubated in 3% hydrogen peroxide solution at room temperature in the dark for 25 min, followed by three washes in phosphate-buffered saline (PBS, pH 7.4) for 5 min each. Tissue sections were blocked with 3% bovine serum albumin (BSA) at room temperature for 30 min before incubation with primary antibodies against CLOCK, OX2R, and 5-HTR1A (1:200 dilution) at 4 °C overnight. The next day, the sections were washed with PBS and incubated with goat anti-rabbit IgG secondary antibodies at room temperature for 50 min. Immunoreactivity was visualized using a DAB detection kit, and cell nuclei were counterstained with hematoxylin. Sections were then rinsed in distilled water, dried, mounted with glycerol gelatin, and air-dried before imaging using an Olympus microscope (Tokyo, Japan). All reagents were sourced from ServiceBio Technology Co., Ltd. (Wuhan, China). Positive staining was indicated by yellowish-brown coloration under the microscope, and protein expression levels were quantified by measuring the integrated optical density (IOD) of positive areas.

### 4.8. RNA Preparation and Sequencing

Following exposure to a simulated 5500 m hypobaric hypoxia environment, the rats were euthanized via intraperitoneal injection of an overdose of 30 g/L sodium pentobarbital. The hypothalamus was rapidly dissected on ice, rinsed in pre-cooled saline, and immediately flash-frozen in liquid nitrogen. Total RNA was extracted, and transcriptomic data were obtained via library construction and sequencing on the Illumina platform (San Diego, CA, USA). Differentially expressed genes (DEGs) were identified based on a fold change (FC) > 1.5 and a *p*-value < 0.05. GO and KEGG pathway enrichment analyses were conducted on the identified DEGs, and results were visualized using bar charts and bubble plots generated via an online platform https://www.bioinformatics.com.cn (18 December 2024).

### 4.9. Enzyme-Linked Immunosorbent Assay (ELISA)

At the end of the experiment, the rats were euthanized as previously described, and the hypothalamus was rapidly isolated. The tissue was weighed and homogenized in PBS at a 1:10 ratio. The homogenate was centrifuged at 5000 rpm for 15 min at 4 °C, and the supernatant was collected. The total protein concentration of each sample was determined using a BCA protein assay kit. ELISA assays were performed using commercial kits according to the manufacturer’s instructions to determine target protein levels.

### 4.10. Western Blot Analysis

Total protein was extracted from the hypothalamus using RIPA lysis buffer supplemented with protease inhibitors, followed by centrifugation at 12,000 rpm for 15 min at 4 °C. Protein concentrations were measured using a BCA assay kit. The protein samples were mixed with loading buffer at a 1:4 ratio and subjected to SDS-PAGE on 7.5–12% polyacrylamide gels. Proteins were transferred onto nitrocellulose membranes via wet transfer. The membranes were blocked with 5% non-fat milk and incubated overnight with primary antibodies against OXR2, 5-HTR1A, CLOCK, CRY2, PER2, and BMAL1 (1:1000 dilution). After three washes with TBST, the membranes were incubated with HRP-conjugated secondary antibodies (anti-rabbit or anti-mouse IgG, 1:5000 dilution). β-Actin was used as a loading control. Protein bands were visualized using a chemiluminescent detection system.

### 4.11. Statistical Analysis

Statistical analysis was performed using GraphPad Prism 8 (GraphPad Software, La Jolla, CA, USA). Data were presented as mean ± standard deviation (x ± s). Comparisons between two groups were conducted using an unpaired *t*-test, while one-way ANOVA was used for multiple group comparisons. Chi-square tests were applied for categorical variables. A *p*-value of <0.05 was considered statistically significant.

## 5. Conclusions

Continuous monitoring of sleep duration in rats under hypobaric hypoxia for 7 days confirmed that while total sleep duration remained unchanged, sleep rhythms were disrupted, exhibiting an initial stress response followed by adaptation. The most pronounced changes occurred on the third day, making it a potential model for high-altitude insomnia. Peripheral blood analysis indicated that hypobaric hypoxia caused damage to cardiac, hepatic, and renal functions.

Serum biomarker analysis revealed that hypoxia-induced oxidative stress was characterized by decreased levels of SOD, CAT, and GSH, accompanied by increased MDA levels. This led to elevated levels of inflammatory cytokines such as IL-1β and IL-6, activating the HPA axis and increasing the secretion of CRH, ACTH, and CORT, ultimately triggering insomnia. Pathological analysis demonstrated hypothalamic tissue damage and increased permeability of the blood–brain barrier, as indicated by elevated S100β levels.

Preliminary transcriptomic analysis identified potential molecular mechanisms underlying sleep disturbances induced by hypobaric hypoxia, highlighting key pathways such as serotonergic synapses, glutamatergic synapses, lipid metabolism, IL-17 signaling, and cortisol synthesis and secretion. Additionally, central nervous system studies revealed increased expression of wakefulness-related neurotransmitters (Glu, DA, and NA) and orexin receptors. Furthermore, hypoxia altered the transcription–translation feedback loop (TTFL), suppressing the expression of positive regulators (CLOCK and CRY2) while upregulating negative regulators (BMAL1 and PER2), ultimately leading to circadian rhythm disruptions in rats.

## Data Availability

The data presented in this study are available on request from the corresponding author (accurately indicate status).
